# Whole-genome analyses reveal a novel prophage and cgSNPs-derived sublineages of *Brachyspira hyodysenteriae* ST196

**DOI:** 10.1186/s12864-022-08347-5

**Published:** 2022-02-15

**Authors:** Ana Belén García-Martín, Thomas Roder, Sarah Schmitt, Friederike Zeeh, Rémy Bruggmann, Vincent Perreten

**Affiliations:** 1 Division of Molecular Bacterial Epidemiology and Infectious Diseases, Institute of Veterinary Bacteriology, Vetsuisse Faculty, University of Bern, Bern, Switzerland; 2 Graduate School for Cellular and Biomedical Sciences, University of Bern, Bern, Switzerland; 3 Interfaculty Bioinformatics Unit and Swiss Institute of Bioinformatics, University of Bern, Bern, Switzerland; 4 Section of Veterinary Bacteriology, Institute for Food Safety and Hygiene, Vetsuisse Faculty, University of Zurich, Zurich, Switzerland; 5 Clinic for Swine, Department of Clinical Veterinary Medicine, Vetsuisse Faculty, University of Bern, Bern, Switzerland; 6 Institute of Veterinary Bacteriology, University of Bern, Länggassstrasse 122, CH-3012 Bern, Switzerland

**Keywords:** Bioinformatics, Horizontal-gene transfer, Pangenome, Structural variations, Singletons, Swine dysentery, WGS

## Abstract

**Background:**

*Brachyspira* (*B.*) *hyodysenteriae* is a fastidious anaerobe spirochete that can cause swine dysentery, a severe mucohaemorragic colitis that affects pig production and animal welfare worldwide. In Switzerland, the population of *B. hyodysenteriae* is characterized by the predominance of macrolide-lincosamide-resistant *B. hyodysenteriae* isolates of sequence type (ST) ST196, prompting us to obtain deeper insights into the genomic structure and variability of ST196 using pangenome and whole genome variant analyses.

**Results:**

The draft genome of 14 *B. hyodysenteriae* isolates of ST196, sampled during a 7-year period from geographically distant pig herds, was obtained by whole-genome sequencing (WGS) and compared to the complete genome of the *B. hyodysenteriae* isolate Bh743-7 of ST196 used as reference. Variability results revealed the existence of 30 to 52 single nucleotide polymorphisms (SNPs), resulting in eight sublineages of ST196. The pangenome analysis led to the identification of a novel prophage, *pphBh*CH20, of the *Siphoviridae* family in a single isolate of ST196, which suggests that horizontal gene transfer events may drive changes in genomic structure.

**Conclusions:**

This study contributes to the catalogue of publicly available genomes and provides relevant bioinformatic tools and information for further comparative genomic analyses for *B. hyodysenteriae*. It reveals that Swiss *B. hyodysenteriae* isolates of the same ST may have evolved independently over time by point mutations and acquisition of larger genetic elements. In line with this, the third type of mobile genetic element described so far in *B. hyodysenteriae*, the novel prophage *pphBh*CH20, has been identified in a single isolate of *B. hyodysenteriae* of ST196.

**Supplementary Information:**

The online version contains supplementary material available at 10.1186/s12864-022-08347-5.

## Background

*Brachyspira* (*B.*) *hyodysenteriae* is a fastidious anaerobe spirochete that can cause swine dysentery (SD), a severe mucohaemorrhagic colitis that affects pig farm industry and animal welfare worldwide [1]. In the last years, SD has been controlled by using i.a. antimicrobial agents of the pleuromutilin, macrolide, lincosamide and tetracycline classes, which has resulted in the selection and emergence of multidrug-resistant *B. hyodysenteriae* strains in many pig producing countries, including Switzerland [1]. Consequently, and considering the reduced arsenal of antimicrobial agents that are authorized and effective against *B. hyodysenteriae*, the treatment and control of SD have turned into new challenges.

As in other bacteria, the *B. hyodysenteriae* genome is evolving through mutations and recombination, paving the way for the formation of new genetic lineages that might have acquired certain advantages for virulence and environmental adaption like surviving antimicrobial exposure. Specific mutations in hemolysin genes have been shown to be associated with weak and strong hemolytic *B. hyodysenteriae* strains [2]. In addition, differential transcriptional patterns underlying different hemolytic phenotypes in *B. hyodysenteriae* have been reported [3].

Concerning antimicrobial resistance in *B. hyodysenteriae*, resistance to ribosomal-targeting drugs of the macrolide, lincosamide and tetracycline classes has been linked to the presence of single point mutations on the 23 S rRNA and 16 S rRNA, respectively [4–8].

Recently, gene transfer into the genome of *B. hyodysenteriae* has been also shown to contribute to antimicrobial resistance. So far, two acquired antimicrobial resistance genes, the lincosamide resistance gene *lnu*(C) and the tiamulin-valnemulin resistance gene *tva*(A), have been identified in *B. hyodysenteriae* [4, 5, 9, 10]. These findings indicate that *B. hyodysenteriae* can acquire antimicrobial resistance genes, such as the *lnu*(C), associated to the transposon MnT*Sag*1 originally found in *Streptococcus agalactiae* by horizontal gene transfer (HGT) mediated by mobile genetic elements (MGEs) [11]. To date, a single MGE, i.e. the defective prophage VSH-1 of *B. hyodysenteriae*, has been shown to mediate intraspecific HGT in *in vitro* experiments, indicating that such gene transfer agent may also play a role in gene acquisition in *B. hyodysenteriae* [12, 13]. MGEs can contribute to the acquisition of elements conveying advantages associated with antimicrobial resistance, virulence and environmental adaption that can be further fixed and spread by clonal expansion [14, 15].

A powerful comparative approach to detect acquired novel MGEs, acquired antimicrobial resistance genes and putative virulence factors is the pangenome analysis [16, 17]. At higher resolution, single nucleotide polymorphisms (SNPs) are usually analysed to understand their contribution to the expansion of both specific clonal lineages and sublineages. In this line, studies focused on bacterial epidemiology, genetic diversity and population structure are frequently based on non-recombinant core genome SNPs (cgSNPs) [18, 19].

In Switzerland, over nearly the last decade, SD has been caused mainly by a specific predominant macrolide-lincosamide-resistant *B. hyodysenteriae* belonging to sequence type ST196 [20], only reported in Swiss pig herds so far [4, 5, 20]. This fact prompted us to perform whole-genome sequencing (WGS), pangenome and SNP analyses to get deeper insights into the genome structure and variability of different ST196 isolates. Our findings shed light on the genetic diversity of *B. hyodysenteriae* ST196 and revealed the presence of the novel prophage *pphBh*CH20 in a single isolate of *B. hyodysenteriae* of ST196.

## Methods

### Isolates information, bacterial culture and DNA extraction

Fourteen isolates of *B. hyodysenteriae* of ST196, isolated from geographically distant Swiss pig herds without known epidemiological links (Additional Fig. S1) between 2010 and 2016, were sequenced in this study and compared to the closed genome of the *B. hyodysenteriae* isolate Bh743-7 (GenBank accession numbers CP046932 (chromosome), CP046933 (plasmid)) of ST196 isolated in 2017 and used as reference [21]. All 15 isolates included in this study, except two (isolates B 114_09C and B 115_02A), were obtained from pigs with SD [20]. All except one, contained the A2058T mutation in the 23 S rRNA associated with the macrolide-lincosamide resistance phenotype [20]. None of the isolates harbored any known acquired antimicrobial resistance genes such as the *lnu*(C) and *tva*(A) [20]. High-quality genomic DNA of *B. hyodysenteriae* was extracted from the bacterial lawn superficially grown on trypticase soy agar plates containing 5% (v/v) sheep blood (TSA-SB, Becton Dickinson), using a DNeasy® Blood & Tissue kit (Qiagen) following the manufacturer’s instructions. For each sample, the bacterial lawn of at least two TSA-SB plates were collected using a 10 µL plastic loop and resuspended in 300 µL of resuspension buffer three times to wash away remnants of material from the agar plates, before continuing with the protocol. All DNA samples were RNAse (20 mg mL^− 1^) treated for 60 min at 37 ºC, purified using AMPure® XP magnetic beads (Beckmann Coulter) and quantified using a Qubit 3.0 fluorometer (Life Technologies).

### Genome sequencing, assembly and annotation

Standard genomic libraries, containing unique dual indexes, were prepared from genomic DNA obtained from all *B. hyodysenteriae* isolates of ST196, including the isolate Bh743-7 for which we had previously generated its complete genome by Oxford Nanopore Technologies sequencing and hybrid assembly (CP046932 and CP046933) [21]. The libraries were sequenced using the Illumina® HiSeq platform (Eurofins Genomics GmbH, Germany) in the sequencing mode NovaSeq^TM^ 6000 2-PE 2 × 150 bp. Short reads were checked for quality using FastQC v0.11.7 [22] and quality control output files were combined into a single report using MultiQC v1.8 [23]. Illumina adapters, nucleotides at both ends with an average Phred score < 15 over a 4 bp sliding window, reads shorter than 36 bp and low quality bases (average Phred score < 33) were removed using Trimmomatic v0.36 [24].

Illumina paired-end short reads were assembled into contigs using the multi-*k*mer *de Bruijn* graph-based assembler SPAdes v3.12 in the *--careful* mode [25]. All draft genomes were filtered for contigs larger than 500 bp with depth coverage above 100X, using a custom Python script (Additional file 1), and their quality was assessed using QUAST v4.6.0 [26]. Genome annotation was done locally using the NCBI-PGAP pipeline v4.12 [27].

### SNP variants analysis of *Brachyspira hyodysenteriae* of ST196

Core genome single nucleotide polymorphisms (cgSNPs) were called using Snippy v4.5 [28], with default parameters, providing the complete genome of the *B. hyodysenteriae* isolate Bh743-7 as a reference genome for WGS alignment. A phylogenetic tree, based on non-recombinant cgSNPs filtered using Gubbins with default parameters [29], was constructed with FastTree [30], and visualized and edited with iTOL v5.7 [31]. The non-recombinant cgSNP alignment was converted into a pairwise distant matrix using snp-dists v.0.7.0 (https://github.com/tseemann/snp-dists). A complementary heatmap displaying distances across genomes was generated with the R package “gplots” (https://github.com/talgalili/gplots). The functional effect of SNPs was investigated by SnpEff v4.3T [32], via Snippy. A custom Python parser script (Additional file 2) was used to combine and extract the information relative to all SNPs that were common to all ST196 isolates.

### Pangenome analysis of *Brachyspira* spp.

Similarity across genomes of *B. hyodysenteriae* of ST196 was also analysed at nucleotide sequence level by calculating whole-genome average nucleotide identity (ANI) scores, using the Python module PyANI v0.2.0 [33] via Anvi’o v6.2 [34].

The pangenome analysis of all *B. hyodysenteriae* ST196 genomes, extended to a total of 90 genomes, including those corresponding to other *B. hyodysenteriae* STs (ST6, ST66 and ST197) circulating in Switzerland and also those belonging to other *Brachyspira* species, was computed using Anvi’o v6.2 [34].

GenBank-formatted public genomes and linked metadata (Additional file 3) were downloaded and processed following a Snakemake [35] workflow in Anvi’o (http://merenlab.org/2019/03/14/ncbi-genome-download-magic/). For downstream analyses, the unpublished NCBI-PGAP annotation files corresponding to the complete genome of *B. hyodysenteriae* isolate Bh743-7 and draft genomes of fourteen additional isolates of ST196 were reformatted prior importation into Anvi’o, using the Bioinformatics Tools (Bit) package v1.4.71 (https://github.com/AstrobioMike/bioinf_tools) [36]. Additional structural and functional annotations for each genome were done using Prodigal [37] and the Cluster of Orthologous Groups (COGs) database [38], respectively, as part of the Anvi’o workflow. A contigs database containing information about contig number, sequence composition, structural and functional annotation was generated for each genome and provided to Anvi’o to generate a genome-storage database using the --*external-genome* flag. Next, a pangenome analysis was computed using the anvi-pan-genome program with parameters --*min-bit* 0.5 (default) and --*mcl-inflation* 10 (recommended for genetically closely related genomes), as in [39]. Genomes were organized based on shared gene clusters using Euclidean distances and Ward linkage, the number of genomes that contributed to each gene cluster (*number of genomes has hits*) and, when required, by forcing synteny. Genes clusters were grouped into bins containing core genes (common to all the isolates), soft-core genes (could be present/absent) and singletons (only present in a single genome), and saved as a default collection for subsequent summary analysis. In addition, a homogeneity index, which takes 1 as highest value and provides an idea of shared sequence identity, was obtained after calculating both functional (amino acid residue conservation without considering sequence gaps) and geometric (sequence gaps and amino acid residue patterns) indexes using Anvi’o (see “An Anvi’o workflow for microbial pangenomics – Meren Lab”). Details on programs and parameters are presented in the additional file 4. Hemolysin genes obtained from the pangenome analysis were screened for presence of mutations described previously [2] by multiple sequence alignment using Clustal Omega and the genome of the *B. hyodysenteriae* WA1 strain (NC_012225.1) as a reference.

### Prophage Hunter analysis

The complete chromosome of *B. hyodysenteriae* isolate Bh743-7 of ST196 (CP046932) was interrogated using the Prophage Hunter web server (https://pro-hunter.genomics.cn/) [40], in order to detect prophage elements by similarity comparison to elements already deposited in a reference database. The probability of a predicted prophage being active was provided by the activity score ranging from 0 to 1.

### Basic alignment, schematic gene map representation and visualization

Basic alignment visualization of the annotated prophage elements and genomic context analyses were based on the application of the progressive algorithm MAUVE v2.4.0 [41]. Comparison of phage-like regions and schematic gene map were done using Easyfig v2.0 [42]. Final figures were edited using the open-source vector graphics editor Inkscape v1.0 (https://inkscape.org/).

## Results

### Genome sequencing, assembly and annotation

The de novo draft assemblies of all isolates of ST196 were obtained through assembly of Illumina paired-end reads using SPAdes v3.12 (Additional Table S1). These assemblies were characterized by a low G+C content (~27.1%) and a variable number of contigs (Additional Table S1). They contained between 25 and 36 contigs larger than 500 bp and with a minimum coverage of 100X. The N50 metric was above 292,250 bp for all assemblies. Assembly lengths ranged from ~3.01 Mbp (BHZ333) up to ~3.07 Mbp (Bh743-7). The average assembly size was 3.04 Mbp reflecting a high quality in terms of completion compared to the complete *B. hyodysenteriae* genomes of the strains WA1 (CP001357.1), B-78^T^ (NZ_CP015910.2), BH718 (CP019600.1) and Bh743-7 (CP046932 and CP046933) publicly available. On average, 2605 total CDS, 2583 coding genes and 40 rRNAs were obtained. Contigs containing plasmid-encoded genes were found in 12 of the 14 additional ST196 isolates using BlastN. Their size ranged between 31,271 and 32,625 bp and their G+C content varied between 22.3% and 22.5%. All draft genomes, including the one without plasmid and the one from which the plasmid could not be reconstructed from a single contig, were further analysed.

### Genetic diversity across closely related genomes of *Brachyspira hyodysenteriae* of ST196

**Fig. 1:**
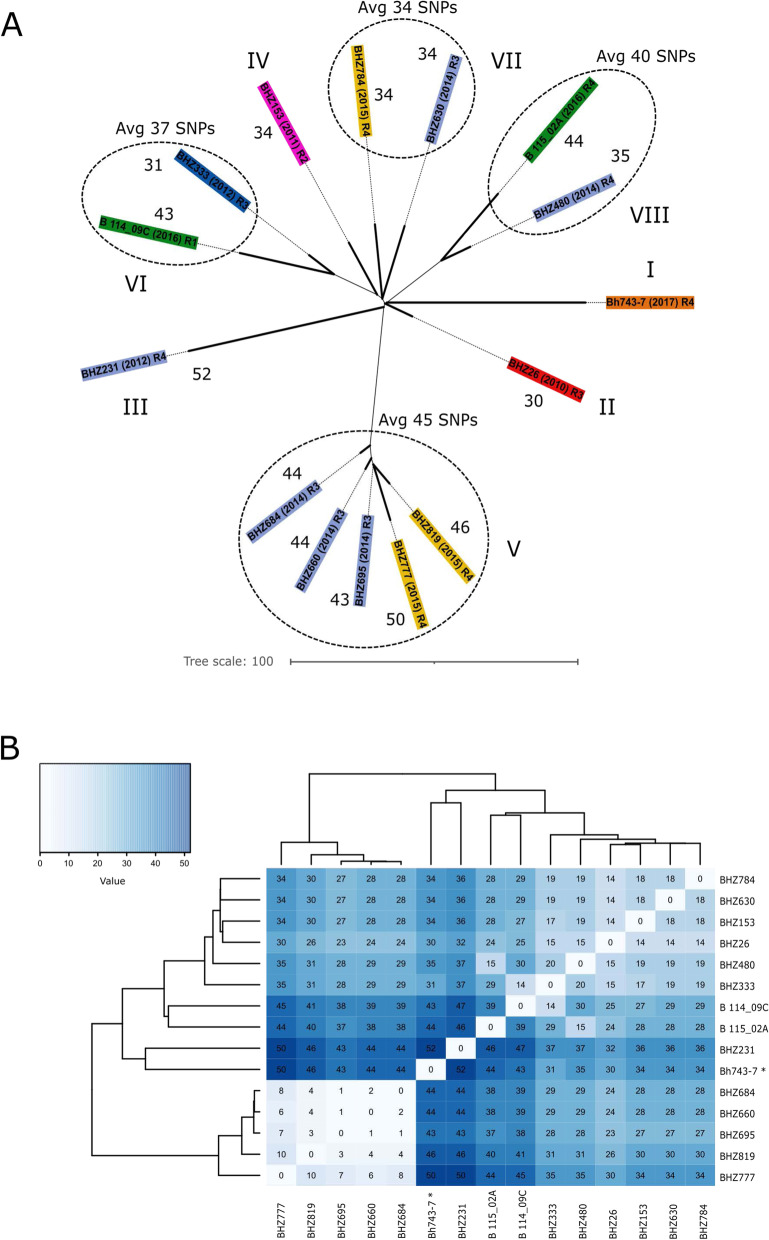
Core-genome SNP-based phylogeny of *Brachyspira hyodysenteriae* isolates of ST196. **A** The relationship among isolates of ST196 are shown according to the approximately-maximum-likelihood phylogenetic tree. Labels containing names of the isolates and regions (from Western to Eastern Switzerland: R1 to R4) of isolation are colored according to the years of isolation. Sublineages are indicated with roman numerals. Numbers of different non-recombinant cgSNPs identified respect to the reference genome are indicated for each isolate. Average (Avg) SNPs distance is also indicated for clusters V, VI, VII and VIII. **B** Isolates are clustered according to the distance matrix of pairwise differences calculated from the non-recombinant cgSNPs. Number of different cgSNPs used to identify genetic distances across all genomes are indicated in each cell of the heatmap

According to the Snippy analysis, on average, the core genome alignment had a length of ~3.02 Mbp (STDEV = 8912 bp; minimum (2,985,653 bp, 96.77% of aligned bases) and maximum alignment (3,020,968 bp, 97.92% of aligned bases)). A total of 153 different non-recombinant cgSNPs were detected from the 15 isolates of *B. hyodysenteriae* of ST196. Pairwise comparisons revealed that the isolates differed by at least one and at most 52 SNPs, generating eight phylogenetic sublineages (I - VIII) (Fig. 1 A and 1 B). These sublineages consisted of either clusters of highly related isolates or singletons differing from the *B. hyodysenteriae* reference isolate Bh743-7 by a minimum of 30 and up to 52 cgSNPs (Fig. 1 B). The singletons (I - IV) consisted of four isolates which were all obtained from different herds in different years (Fig. S1). With respect to the reference isolate Bh743-7 (from 2017), the isolate BHZ26 (2010) differed by 30 SNPs, and the isolate BHZ153 (2011) by 34 SNPs. The most divergent isolate (BHZ231), sampled in 2012, harboured 52 different SNPs. The average SNP distance calculated for the isolates contained in clusters V-VIII was 41 SNPs (with a minimun of 31 SNPs and a maximum of 50 SNPs) (Fig. 1 A). Cluster V contained five isolates differing by a minimun of 43 SNPs and a maximum of 50 SNPs (average SNP distance of 45 SNPs). Three of them (BHZ660, BHZ684, BHZ695) were highly related (one to two SNPs, Fig. 1 B) and were isolated in 2014 from three different pig herds. The other two (BHZ777, BHZ819), differing by 10 SNPs, were sampled from two different pig herds in 2015, and differed by a minimum of three and up to eight SNPs from the previous isolates (Fig. 1 B and Fig. S1). Each of the three remaining clusters (VI - VIII) contained two isolates sampled in different years from several herds. The isolates of these clusters differed by a minimum of 31 SNPs and a maximum of 44 SNPs (average SNP distance of 37 SNPs) compared to the reference genome (Fig. 1 A and 1 B). Overall, sublineages conformation was not associated neither by year nor region of isolation.

Regarding the variants, 26 were found in all ST196 that were compared to the reference genome (Additional file 5). Of these, 23 were found in protein-coding sequences and the remaining three in non-coding sequences. From the 23 SNPs in protein-coding sequences, 20 were translated into non-synonymous including one classified as frameshift, three as stop-lost, and six as missense. The missense SNPs were found in genes involved in chemotaxis, transport and metabolism of ions, carbohydrate and inorganic substrates, cell wall/membrane/envelope biogenesis, signal transduction, transcription and translation. In addition, carbon starvation proteins and different enzymes, involved in general metabolism pathways, were represented among the variants-containing genes (Additional file 5).

### High conservation of the core genome in *Brachyspira hyodysenteriae*

**Fig. 2:**
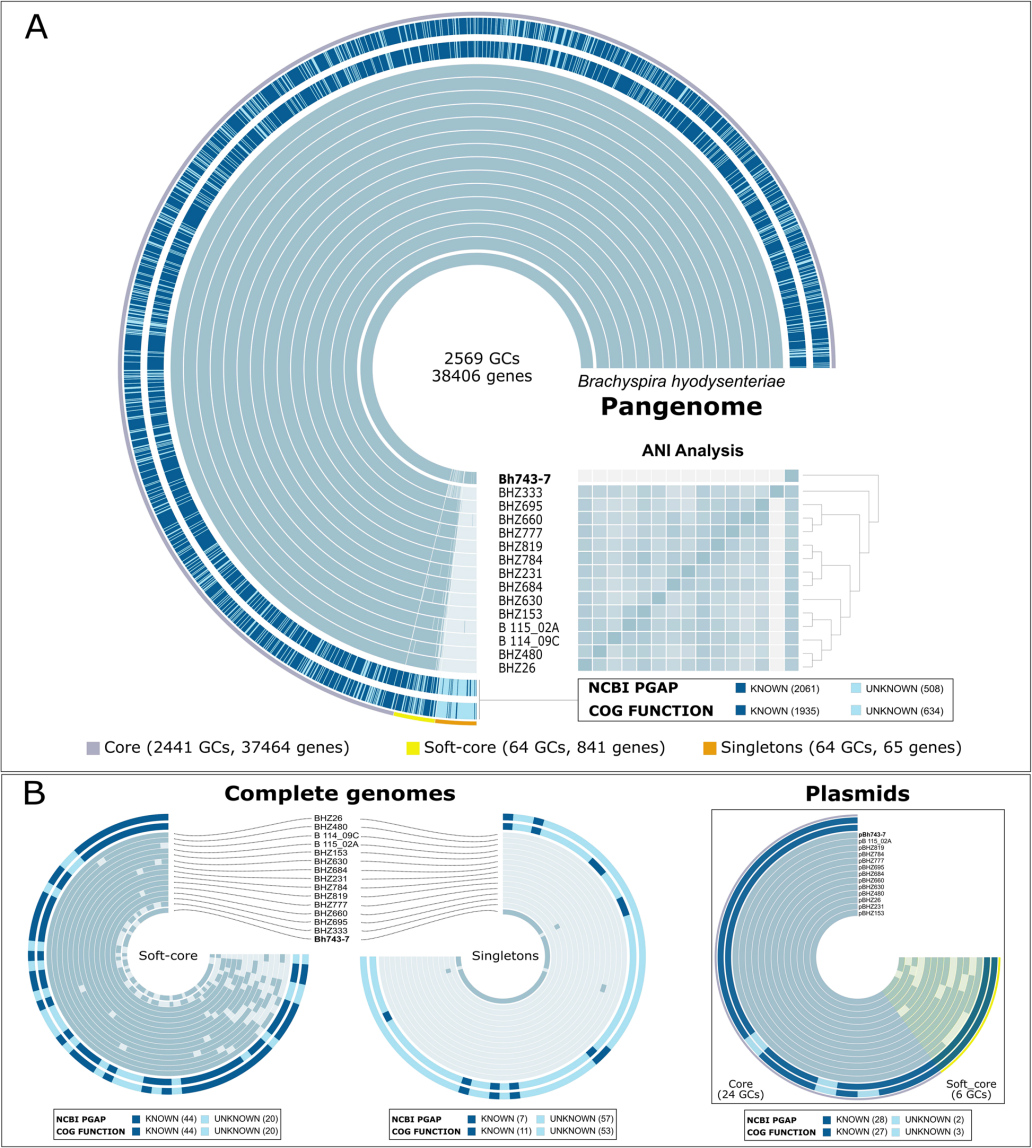
Pangenome analysis of *Brachyspira hyodysenteriae* isolates of the predominant ST196. Total number of genes as well as bin-specific gene clusters (GCs) are indicated. **A** The two outmost layers represent both functional annotations derived from the COGs database and NCBI-PGAP. Known and unknown functions are in dark and light blue, respectively. The number of either known or unknown functions are indicated in brackets. Each blue-colored layer represents the genome of a *B. hyodysenteriae* isolate of ST196. The inner one represents the complete genome of *B. hyodysenteriae* isolate Bh743-7 used as reference genome. Genomes are organized by average full nucleotide identity (ANI values: 0.995 − 1) and minimum number of genomes in which a certain gene is present. Core (grey), soft core (yellow) and singletons (orange) bins are shown for all the genomes (n = 15). **B** Soft-core and singleton bins are represented in more detailed at the complete genome level (n = 15), but also for the plasmids (n = 13), independently

As shown by the previous cgSNPs and Anvi’o pangenome analyses, the genomes of the ST196 isolates mainly differed from each other by SNPs, being nearly identical throughout their entire lengths (ANIb scores above 99%) (Fig. 2 A). However, lower ANIb scores (97 and 98%) were obtained when the shortest and largest genomes of *B. hyodysenteriae* isolates BHZ333 and Bh743-7 were considered for pairwise comparison (Fig. 2 A). The first pangenome analysis was computed considering the whole set of coding genes of the 15 Swiss isolates of ST196 analysed, revealing the presence of core, soft-core and singletons gene clusters (Fig. 2 A and 2 B). A total of 2569 gene clusters (GCs) containing 38,406 genes were binned into core (2441 (95%) GCs, 37,464 genes), soft-core (64 (2.5%) GCs, 841 genes) and singletons (64 (2.5%) GCs, 65 genes) (Fig. 2 A). The plasmids were nearly identical and contributed to the pangenome with only 30 GCs; of those, 24 were core GCs and six were soft-core GCs. No singletons were identified (Fig. 2 B). Most of the plasmid-encoded genes were classified as players of cellular processes and signalling, followed by transport and metabolism of coenzymes, nucleotides and carbohydrates and motility (Additional file 6). Only two GCs that contained uncategorized genes were found in both bins core and soft-core GCs of the plasmids (Additional file 6). At the complete genome level, out of the 2569 GCs, 2489 were shared among 14 isolates and 2505 GCs were shared by a maximum of four isolates. COGs functions and categories, among which cellular and signalling processes, amino acid transport and metabolism and poorly characterized categories were the most abundant, were assigned to each protein-coding gene (Additional file 6). All eight hemolysin genes were identical at the DNA level and were classified within the core genome (Additional file 6). Four, two and one non-synonymous mutations were detected in the hemolyin III, hemolysin and hemolysin activation protein encoding genes, respectively. The soft-core bin contained 64 GCs, of which 48 were shared among 14 isolates. Forty-two of these GCs were categorized mainly as cellular and signalling genes, but also as poorly characterized ones (Additional file 6). The other 16 GCs were present in a varying number of isolates, and 11 of them contained proteins annotated as acetyl and glycosyl transferases, HAMP domain-containing proteins, radical SAM proteins, alpha-1,2-fucosyltransferase, tetratricopeptide repeat-containing protein and methyl accepting chemotaxis proteins. Concerning the singleton bin, three singletons were found in *B. hyodysenteriae* isolates BHZ660, BHZ695 and B 115_02A (Fig. 2B). Sixty-two singletons were present exclusively in the chromosome of *B. hyodysenteriae* isolate Bh743-7 (Fig. 2 A and 2 B). While most singletons did not have functional annotation, 13 of them were annotated as phage-like proteins (Additional file 6). Among these phage-like genes, only seven were classified into the COGs categories cellular and signalling processes and defense mechanisms (Additional file 6). The organization of the gene clusters based on the synteny of the complete genome of *B. hyodysenteriae* isolate Bh743-7 revealed that these phage-like elements were located in the same chromosomal region (Additional Fig. S2).

### A novel prophage integrated in the genome of *Brachyspira hyodysenteriae* Bh743-7

**Fig. 3:**
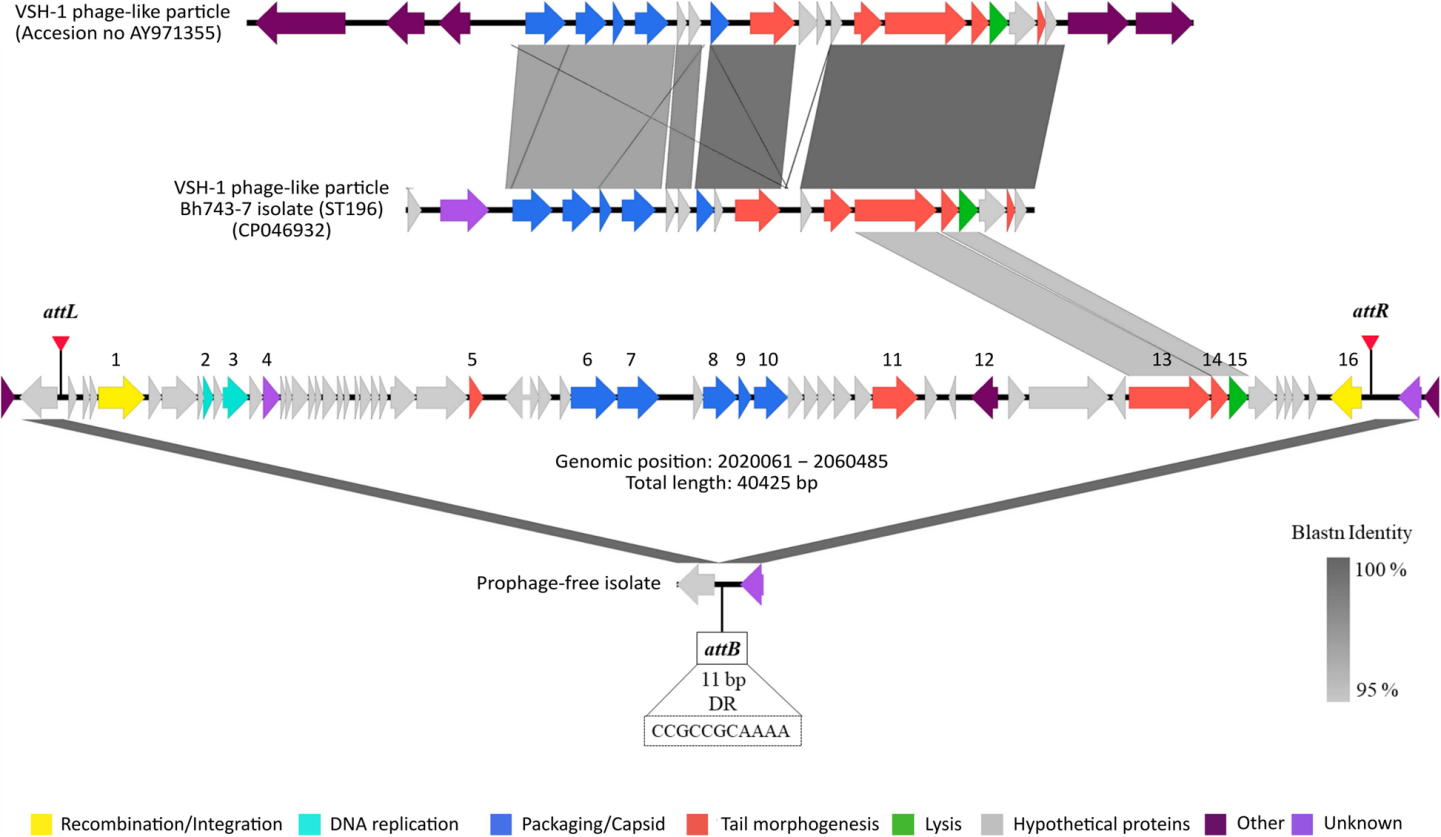
Schematic gene map displaying the modular organization of the novel prophage and its integration into the chromosome of the prophage-carrying *Brachyspira hyodysenteriae* isolate Bh743-7 of ST196. Image was created using Easyfig v2.1 (Sullivan et al., 2011). Each arrow represents an open reading frame and its orientation. Phage-like genes annotations are as follows: 1: Phage recombination protein Bet; 2: Winged helix-turn-helix transcriptional regulator; 3: N-6 DNA-methylase; 4: DUF4406 domain-containing protein; 5: Phage tail protein; 6: PBSX family phage terminase large subunit; 7−10: Phage capsid protein; 11: Phage tail protein; 12: Damage-inducible protein D; 13−14: Phage tail proteins; 15: Glycoside hydrolase family 19 protein; 16: Integrase. Phage-like genes are color-coded according to their respective modules: DNA replication (light blue), packaging/capsid morphogenesis (dark blue), tail morphogenesis (orange), lysis (green), recombination/‌integration (yellow), other functions (purple), unknown functions (violet) and hypothetical proteins (grey). Direct comparison of the structures of the new prophage and the VSH-1 gene transfer agent of *B. hyodysenteriae* is also shown

The analysis of the chromosome of *B. hyodysenteriae* isolate Bh743-7 using Prophage Hunter revealed the presence of a 40,425 bp insert, which corresponded to the phage-like region mentioned above (Fig. 3). This insert was compatible with a predicted active prophage, with an activity score above 0.8 and was characterized by a low G+C content (27.4%). The modular organization of its open reading frames resembled the structure of tailed and double-stranded DNA bacteriophages of the *Siphoviridae* family (e.g. *Streptococcus agalactiae* phage LYGO9 (JX409894)). By comparing the insert-containing isolate Bh743-7 with insert-free isolates of the same ST, we could identify a novel prophage, named *pphBh*CH20. This prophage was integrated in the chromosome between positions 2,020,061 and 2,060,485, between a hypothetical protein and a DUF-domain containing protein, and was flanked by two 11 bp direct repeats (DR) (5′-CCGCCGCAAAA-3′) (Fig. 3). The prophage *pphBh*CH20 contained 58 genes of which 16 were annotated as phage-like genes and the rest as hypothetical proteins (Fig. 3). The 16 annotated genes consisted of one phage recombination protein Bet, one winged helix-turn-helix transcriptional regulator, one N-6 DNA-methylase, one DUF4406 domain-containing protein, three phage tail proteins, one PBSX family phage terminase large subunit, five phage capsid proteins, one damage-inducible protein D, one glycoside hydrolase family 19 protein and one integrase, which were organized in different modules according to their function (Fig. 3). Comparative analysis of the nucleotide sequences of *pphBh*CH20 and VSH-1 (AY971355) revealed that, with an alignment coverage above 99%, three ORFs of the new prophage shared more than 93% sequence identity with protein-coding genes of VSH-1 (Fig. 3). Specifically, two tail proteins and the glycosidase hydrolase (lysin) family 19 protein differed from both Hvp101 and Hvp28 tail proteins and the lysin of VSH-1 by only 32, 11 and six amino acids, respectively (Fig. 3).

**Fig. 4:**
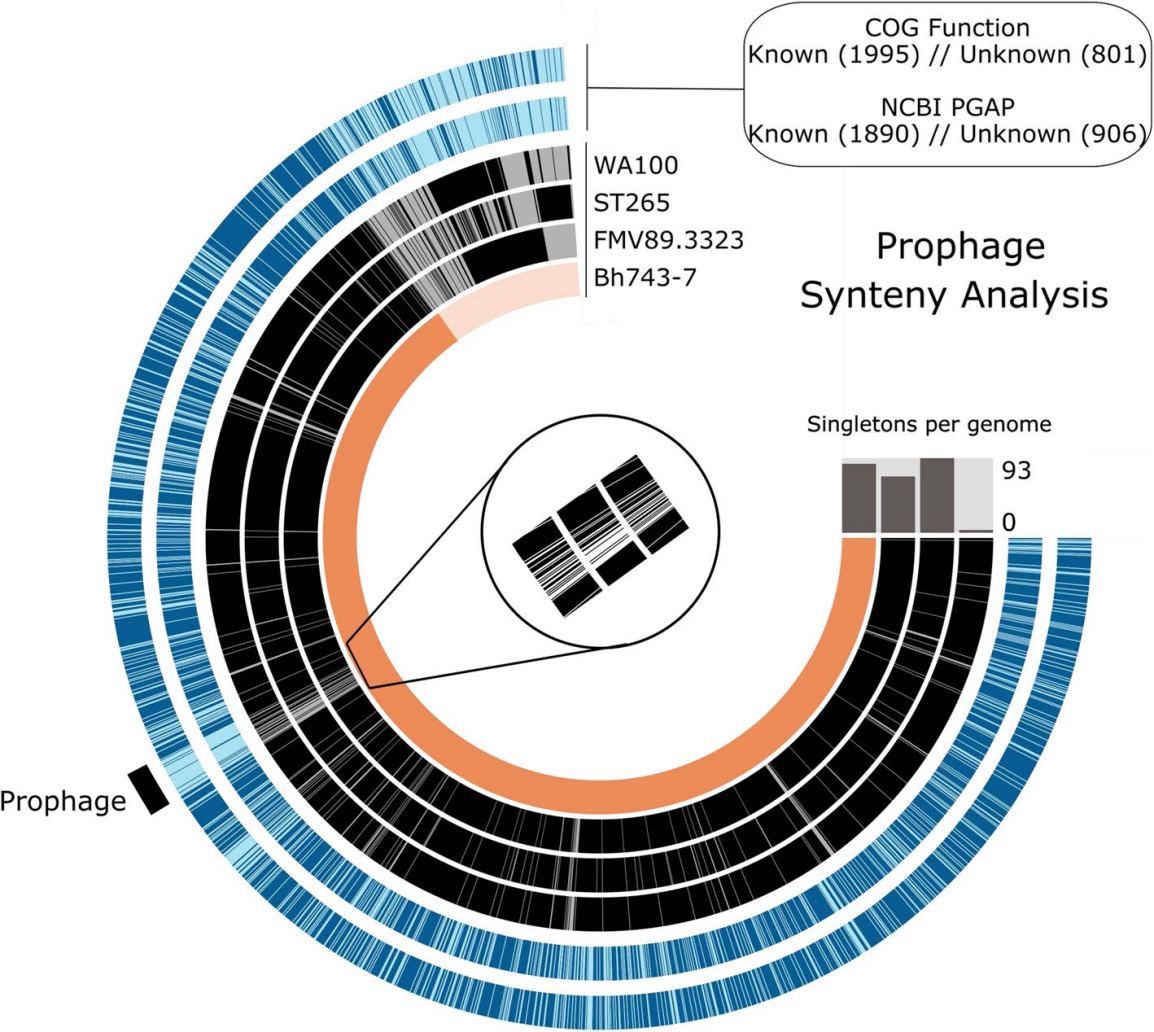
Prophage synteny analysis. The two most outer layers represent both functional annotations derived from the COGs database and NCBP-PGAP. Number of genes with either known (dark blue) or unknown (light blue) annotations are also shown in brackets. The genomes of *Brachyspira hyodysenteriae* strains that share some of the phage-like genes found in the novel prophage of *B. hyodysenteriae* isolate Bh743-7 (in orange) are represented by the black inner layers. Although the novel prophage was not present in any of the analysed strains, homologous genes were found in those strains by forcing synteny as highlighted. In the centre, a zoom in barcode-like graph is showing the presence (black) /absence (white) of the homologous genes. Total number of singletons per genome is also indicated in the bar graph

To determine whether other *Brachyspira* carried the novel prophage *pphBh*CH20 of *B. hyodysenteriae* isolate Bh743-7, as well as other new prophages, a pangenome analysis was conducted with additional *Brachyspira* species genomes (Additional Fig. S3). More than half of the genomes used in this analysis belonged to the species *B. hyodysenteriae* (n = 54). The others were from *B. aalborgi* (n = 17), *B. pilosicoli* (n = 8), *B. hampsonii* (n = 7), *B. murdochii* (n = 2), *B. intermedia* (n = 1), and *B. suanatina* (n = 1). The new pangenome analysis comprised 7401 GCs containing 225,366 gene calls, of which more than 50% had neither COGs nor NCBI-PGAP functional annotations (Fig. S3). In general, *B. hyodysenteriae* genomes had more functional annotations assigned than other *Brachyspira* species genomes (Additional file 7). Most genes were not shared by all genomes and were binned into either soft-core (5029 GCs, 68.0%) or singleton (1556 GCs, 21.0%) bins. Singletons were less abundant in the *B. hyodysenteriae* genomes than in those of the other *Brachyspira* species. Less singletons in *B. hyodysenteriae* may arise from the greater number of genomes available for analysis. The core genome represented by all the genes shared among all *Brachyspira* genomes comprised only 816 GCs (11%) containing 76,427 gene calls, many of them with unknown function. Functional annotation of soft-core genes revealed the presence of phage-like genes in the genomes of different *Brachyspira* species. Although containing a high number of singletons without assigned function, the singleton bin was enriched in genes associated with CRISPR/Cas system, endonucleases, transposases, cell wall and lipopolysaccharide synthesis, outer membrane protein, proteases, efflux pump, and transcriptional regulators (Additional file 7). Some phage-like genes were identified in *B. intermedia* strain PWS-A (NC_017243.1), and *B. pilosicoli* strains SP16 (NZ_AFQM01000000.1), B2904 (NC_018607.1), and WesB (NC_018604.1), exclusively. The genome of *B. hyodysenteriae* isolate Bh743-7 contributed to the singleton bin with only 10 genes, eight of which had no functional annotation. The other two annotated genes represented the phage capsid protein and the phage recombination protein Bet of the novel prophage *pphBh*CH20. The reduction in the number of singletons counted for the genome of isolate Bh743-7 suggested the existence of elements shared with other genomes. For instance, prophage elements highly similar (combined homogeneity index ranging from 0.65 to < 1), or even identical (combined homogeneity index equal to 1), to those found in *pphBh*CH20 were also identified, in a varying number, in the genomes of different *Brachyspira* species including *B. hyodysenteriae*, *B. intermedia, B. pilosicoli*, *B. hampsonii*, *B. murdochii*, and *B. aalborgi* (Additional file 7). However, variations in amino acids (see functional homogeneity index) and protein sequence length (see geometric homogeneity index) were observed (Additional file 7). The highest number of homologous phage-like genes was shared among *B. hyodysenteriae* strains FMV89.3323 (JXNB00000000.1), ST265 (NZ_JXNQ00000000.1), WA100 (NZ_JXNS00000000.1) and Bh743-7 (CP046932 and CP046933). Despite those elements seem to be part of a prophage region, as shown by forcing synteny according to the gene organization of the complete genome of *B. hyodysenteriae* isolate Bh743-7, they co-occurred randomly and synteny was only partially resembled (Fig. 4). The complete novel prophage *pphBh*CH20 was not found in any of the other genomes analysed here, but the results indicated that other prophages or parts of them are present in *Brachyspira* spp. (Additional file 7).

## Discussion

This study presents a unique comparative analysis of 15 genomes of *B. hyodysenteriae* belonging to the same ST and reveals different sublineages, as well as a new prophage. All assemblies obtained here were of high quality in terms of completeness (as compared to the ST196 reference genome) and accuracy (supported by high depth of coverage and high similarity among sequences at both nucleotide and protein sequence levels). All genomes consisted of a pair of replicons corresponding to one chromosome and one plasmid in concordance with the known structure of *B. hyodysenteriae* genomes [4, 5, 21, 43–45], except for one genome that lacked the plasmid and another for which the plasmid could not be fully reconstructed. The genomes were characterized by a low G+C content (~27.0%) and a high frequency of long homopolymeric regions and tandem repeats, features that complicate the sequencing process [46].

Despite the fragmentation and inherent limitations of the draft assemblies of the genomes sequenced by Illumina reads, they were similar in the number of contigs and length. Compared to other *B. hyodysenteriae* genomes [47], our nearly complete assemblies gained quality in terms of reduced number of contigs and increased contiguity thanks to advances in sequencing technology and assemblers. Complete plasmids with sizes of ~32 kb could be reconstructed from single contigs in 12 isolates. While the plasmid of the isolate B 114_09C could be reconstructed from two contigs, no plasmid was found in the isolate BHZ333. The role of this *B. hyodysenteriae* plasmid is still not well understood. The absence of plasmid has been reported in some *B. hyodysenteriae* strains not associated with SD [47–49]. In a follow-up publication, it was reported that the absence of four plasmid-encoded genes was predictive of a reduced pathogenic potential in *B. hyodysenteriae* [50]. However, in our study, the isolate BHZ333 lacking the plasmid was obtained from a pig with SD [20], while the isolate B115_02A, harboring all the plasmid-encoded genes described previously [50], was isolated from a pig in which SD was not diagnosed [20]. Previous studies associated pathogenicity with the presence of other chromosomal virulence genes (e.g. ankyrin proteins, outer membrane proteins, proteins associated with chemotaxis and motility, and hemolysins) [43, 51, 52]. Moderately to weakly hemolytic strains of *B. hyodysenteriae* have been reported in different countries [2, 3, 53, 54]. All eight hemolysin genes regarded as important in the pathogenesis of SD [3] were identified in all isolates of ST196 including in the two isolated from pigs with subclinical infections. The two genes known to be associated with the strong hemolytic phenotype were also present [2]. Although none of the mutations reported by Card and collaborators [2] were identified, several non-synonymous mutations were detected in three hemolysin genes. Regarding the hemolytic phenotype of all 15 *B. hyodysenteriae* isolates of ST196, not only changes in both strength and extension of hemolysis, but also loss of hemolytic activity were observed even for a single isolate following repeated culture passages. Whether such phenotypic differences were due to differential gene expression and/or post-transcriptional events, as suggested previously [2, 3], remain unclear and require further investigations. While our data suggest that pathogenic isolates generally harbor intact plasmids, as well as all the eight hemolysin genes, we consider that we are still far from understanding all genetic and environmental factors that orchestrate both mild and full pathogenicity in *B. hyodysenteriae*. Our comparative analysis of 15 genomes of *B. hyodysenteriae* of ST196 provides new insights into the genomic structure and variability within *B. hyodysenteriae* isolate of a same ST over time. Despite observing a high degree of genomic stability, supported by pangenome and ANI analyses, cgSNPs analysis revealed genetic differences across the genomes of *B. hyodysenteriae* isolates of ST196 and the presence of sublineages within the same ST. Most of such differences occurred randomly. Those frameshift, stop-lost, or missense mutations that occurred in protein-coding sequences have not been investigated for change in function.

An association between the different sublineages and both date and region of isolation was not found, suggesting that the different lineages of ST196 have evolved in an independent manner and persisted in Switzerland over nearly a decade. Finding isolates of ST196 over time suggests not only the existence of few common sources, as previously thought [20], but points out towards other factors that should be considered for successful control of SD in Switzerland (e.g. herd management, transportation and biosecurity practices) [55]. Furthermore, the fact of having found macrolide-lincosamide resistance isolates of ST196 being predominant over other STs (ST6, ST66 and ST197) in Switzerland, could be associated with the acquisition and widespread dissemination of point mutations linked to the decreased susceptibility to such antimicrobials [20]. Although WGS and cgSNPs analyses have been used for high resolution comparison and outbreak investigations of other pathogenic bacterial species, such as *Klebsiella pneumoniae* [56, 57], this combination of analyses has only been recently applied to characterize and to assess persistence of *B. hyodysenteriae* [9]. Specifically, it was found that *B. hyodysenteriae* isolates of various STs can persist over time. Previously, persistence of *B. hyodysenteriae* isolates was assessed by multilocus sequence typing analyses. In fact, *B. hyodysenteriae* isolates of ST56, to which the *B. hyodysenteriae* type strain B-78^T^ (NZ_CP015910.2) belongs, were shown to persist in North America over long periods of time [58]. Nonetheless, the authors did not report the existence of sublineages within the same ST56, probably due to the less resolutive power of their seven-housekeeping-genes-based multilocus sequence typing analysis, (see La and collaborators [59]) compared to both WGS and cgSNP analyses. Also supporting our findings, minor differences across Australian *B. hyodysenteriae* isolates within the same ST as well as persistence of certain STs over time were reported in 2016 [60]. Nonetheless, such isolates displayed different antimicrobial susceptibility profiles that were not observed in our previous study [20].

At the pangenome level, considering both core and singleton bins, our results also revealed a highly conserved but flexible genomic structure that can be shaped by the integration of larger genetic elements, such as the novel prophage *pphBh*CH20 found in the chromosome of a single *B. hyodysenteriae* isolate of ST196. The 40,425 bp *pphBh*CH20 constitutes the third different type of mobile genetic element described so far in *B. hyodysenteriae*. It resembles the structure of the tailed and double-stranded DNA *Streptococcus agalactiae* phage LYGO9 (JX409894) of the *Siphoviridae* family [61]. In line with this, the *lnu*(C)-carrying transposon MTn*Sag*1 recently detected in *B. hyodysenteriae*, was originally identified in *S. agalactiae* [11], demonstrating the capability of *B. hyodysenteriae* to acquire foreign genetic material from other bacterial species through various HGT mechanisms [4, 5, 62]. Although other prophages have been identified in *B. intermedia*, *B. murdochii* and *B. pilosicoli* [48, 63], to our knowledge, only the defective prophage VSH-1 has been shown to play a role in HGT in *B. hyodysenteriae* in *in vitro* experiments [62]. Compared to the 16.3 kb genome of VSH-1, which also exhibits the structure of bacteriophages of the *Siphoviridae* family [62], the new prophage *pphBh*CH20 was more than double in size and carried early function genes involved in DNA replication (i.e. N-6 DNA-methylase and winged helix-turn-helix transcriptional regulator). Moreover, three late function genes (two tail proteins and a glycosidase hydrolase family 19 protein) were shared between *pphBh*CH20 and VSH-1, a phenomenon also observed in *Brachyspira* species phages that suggests that VSH-1 is responsible for HGT [63]. So far, a possible role of *pphBh*CH20 in the *B. hyodysenteriae* isolate Bh743-7 could not be elucidated based on the genes present within its structure, since most of the non-phage related genes coded for hypothetical proteins. The fact that this prophage was found in a single Swiss *B. hyodysenteriae* isolate sampled in 2017 could suggest a recent integration event. However, it seems not to provide any advantage on persistence over time so far.

By extending the pangenome analysis to other *Brachyspira* species, we have shown that many genes still remain poorly characterized, as previously reported [48], thus, needing further investigation. The lack of functional annotation clearly reflects the difficulties (e.g. specialized growth requirements, absence of means for genetic manipulation) associated with studies on the slow-growing *Brachyspira* [64]. Despite this absence of information, the generated pangenome data set may serve as a basis for identifying candidate acquired genes that could play a role in antimicrobial resistance like e.g. those coding for putative MATE family efflux transporter and glyoxalase/bleomycin resistance protein/dioxygenase superfamily protein which were present as singletons in specific strains (Additional file 7). Also we were able to identify phage-like genes, likely belonging to phages already described in other genomes of *B. intermedia*, *B. murdochii* and *B. pilosicoli* [48, 63]. Nevertheless, although identical and highly similar genes to those phage-like genes of the novel prophage *pphBh*CH20 were found in three other *B. hyodysenteriae* strains, the prophage structure was not resembled entirely. Of note, these *B. hyodysenteriae* strains were found in Europe (*B. hyodysenteriae* of ST265), Australia (*B. hyodysenteriae* WA100) and Canada (*B. hyodysenteriae* FMV89.3323). It has been shown that bacterial strains can develop different strategies to avoid phage infection and consequently, turn into phage-resistant bacterial strains [65]. Whether all the *B. hyodysenteriae* strains sharing the highest number of homologous phage-like genes are more sensitive to phage infections compared to other strains remains unclear. Alternatively, potential technical limitations that apply to phage genomics (e.g., sequencing process, viral genome assembly and bioinformatics analyses) could compromise the possibility of finding either such phage-like genes or entire prophages [66]. Likely, WGS of *B. hyodysenteriae* strains using long-read sequencing platforms, as well as improvements in phage-like genes annotations, will help to explain our observations. In agreement with other authors [13, 48], the identification of different phage-like genes in different *Brachyspira* species suggests that prophages might be more common than previously thought and that horizontal gene transfer events mediated by prophages play an important role in *Brachyspira* biology and evolution.

## Conclusions

With this study we contribute to the *B. hyodysenteriae* genomes pool with 14 new genomes. Moreover, we highlighted the power of WGS-based analyses to identify genetic differences across *B. hyodysenteriae* isolates within the same ST. Regarding the variability of Swiss *B. hyodysenteriae* of ST196, this in-depth WGS analysis revealed the existence of different sublineages that seem to have evolved independently and persisted in Switzerland over nearly a decade. The implications of such findings need to be considered for epidemiological projects aiming to trace back specific clones to successfully control SD in Switzerland. Moreover, we showed how horizontal gene transfer events in *Brachyspira* spp. can be detected by pangenome analyses, and found a novel prophage *pphBh*CH20 integrated into the chromosome of the *B. hyodysenteriae* isolate Bh743-7. This study paves the way for further research into WGS comparative analysis and into the functionality of the highly abundant poorly characterized genes of *Brachyspira* spp., as well as into phage diversity and phages-*Brachyspira* species interactions.

## Supplementary Information

**Additional file 1: **This file contains python instructions.

**Additional file 2: **This file contains python instructions.

**Additional file 3: **This file contains BioProjects and information of all analysed genomes.

**Additional file 4: **This file contains the commands followed for pangenome analysis using Anvi'o v6.2.

**Additional file 5: **This file contains information regarding core genome single nucleotide polymorphisms, their location and their effect.

**Additional file 6: **This file contains detailed information about the genes that represent the pangenome of* Brachyspira hyodysenteriae* of sequence type ST196.

**Additional file 7: **This file contains detailed information about the entire set of genes that constitute the *Brachyspira* pangenome at the genus level.

**Additional file 8:** **Figure S1.** Geographical distribution of *B. hyodysenteriae* isolates across Switzerland overtime. Each diamond represents one isolate sampled per herd. Different colors are used to identify samples obtained from geographically distant Swiss pig herds in different years between 2010 and 2017. The basemap indicating density of pig population (grey rings) was obtained from the Federal Statistical Office (http://www.bfs.admin.ch), ThemaKart.

**Additional file 9:** **Figure S2. **Synteny analysis. The two most outer layers represent both functional annotations, derived from the COGs database and NCBI-PGAP, and number of genes with either known (dark blue) or unknown (light blue) annotations are also shown. Genomes are organized according to the synteny of the reference genome (in orange). The genome of the novel prophage consisting of phage-like genes organized sequentially and integrated into the chromosome is highlighted in black. Total number of gene clusters (GCs) and genes are indicated for core (dark blue), soft-core (light pink) and singleton (dark pink) bins.

**Additional file 10:** **Figure S3. **Pangenome analysis of genomes of different *Brachyspira* species. (A) General organization and visualization of 90 *Brachyspira* genomes based on the presence/absence of genes and their contribution to the bins core, soft-core and singleton. Total number of gene clusters (GCs) as well as the number of gene calls falling into each bin are shown in brackets. A graph bar displaying the number of singletons per genome, varying from 0 to 174, is also included.The two outmost layers represent both functional annotations derived from the NCBI-PGAP and COGs database. Known and unknown functions are in dark and light blue, respectively. Total number of both known and unknown functions are indicated in brackets. Genomes are colored-coded according to the seven different species they belong to except Swiss *B. hyodysenteriae* genomes that are colored in orange to facilitate their visualization. (B) Visualization of the soft-core gene clusters containing a high number of accessory genes. (C) Singletons present in each genome are highlighted and information regarding functional annotation is indicated in brackets.

**Additional file 11:** Alignment scores of *B. hyodysenteriae* ST196 isolates obtained with Snippy.

**Additional file 12: Table S1.** Metrics and genetic features of the de novo draft assemblies of *B. hyodysenteriae* isolates of ST196. File containing a table with metrics and genetic features of the de novo draft assemblies of 15 *B. hyodysenteriae* of ST196.

## Data Availability

All supporting data, scripts and workflow commands are provided as additional material. Raw sequencing datasets are deposited at the SRA database and accessible under SRA study SRP306907. Genome assemblies obtained in this study belong to the BioProject PRJNA697976 and are available under the accession numbers JAFCST000000000 (BHZ26), JAFCSS000000000 (BHZ153), JAFCSR000000000 (BHZ231), JAFCSQ000000000 (BHZ333), JAFCSP000000000 (BHZ480), JAFCSO000000000 (BHZ630), JAFCSN000000000 (BHZ660), JAFCSM000000000 (BHZ684, JAFCSL000000000 (BHZ695), JAFCSK000000000 (BHZ777), JAFCSJ000000000 (BHZ784), JAFCSI000000000 (BHZ819), JAFCSV000000000 (B 114_09C) and JAFCSU000000000 (B 115_02A). Accession information is also provided for each genome assembly retrieved from GenBank as additional material (see additional file 3).
