# Supplementary material for: Whole-genome analyses reveal a novel prophage and cgSNPs-derived sublineages of Brachyspira hyodysenteriae ST196
Source: BMC Genomics. 2022 Feb 15;23:131. doi: 10.1186/s12864-022-08347-5 (PMC8845278; doi:10.1186/s12864-022-08347-5)
Supplement: Supplementary file 4 — Additional file 4: This file contains the commands followed for pangenome analysis using Anvi'o v6.2. [file 12864_2022_8347_MOESM4_ESM.docx]

**Anvi′o v6.2_Workflows**

**General pangenome analysis workflow**

**A.1. GenBank formatted unpublished NCBI_PGAP annotation files**

(See original workflow in <http://merenlab.org/2016/11/08/pangenomics-v2/>

$:conda activate base

$:bit-genbank-locus-clean-slate -i annotation.gbk -o reformat_annotation.gbk

(see <https://github.com/AstrobioMike/bioinf_tools>)

$: conda activate anvio-6.2

$:anvi-script-process-genbank -i reformat_annotation.gbk --output-gene-calls gene_calls.tsv --output-functions functions.tsv --output-fasta assembly.fasta --annotation-source NCBI_PGAP

$:anvi-gen-contigs-database -f assembly.fasta -o contigs.db -n assembly --external-gene-calls gene_calls.tsv

$:anvi-import-functions -c contigs.db -i functions.tsv

$:anvi-run-ncbi-cogs -c contigs.db -T7

$:anvi-run-hmms -c contigs.db -T7

- Prepare an external-genomes.txt (2 columns file: sample name and contigs_db_path)

$:anvi-gen-genomes-storage -e external-genomes.txt -o GENOMES.db --gene-caller NCBI_PGAP

$:anvi-pan-genome -g GENOMES.db -n Pangenome --mcl-inflation 10 -T7

$:anvi-display-pan -p Pangenome/*PAN.db -g GENOMES.db

Create collection of bins containing core (--min-num-genomes-gene-cluster-occurs (INTEGER: total number of genomes to be considered)), singletons (--max-num-genomes-gene-cluster-occurs (INTEGER: 1) and soft-core (rest of the genes) using the interactive display and save it as default for further analysis (get summary).

$:anvi-split -p Pangenome/*PAN.db -g GENOMES.db -C default -o SPLIT_PANs

$:anvi-display-pan -p SPLIT_PANs/CORE/PAN.db -g GENOMES.db

$:anvi-display-pan -p SPLIT_PANs/SOFT_CORE/PAN.db -g GENOMES.db

$:anvi-display-pan -p SPLIT_PANs/SINGLETONS/PAN.db -g GENOMES.db

Convert .svg images to .png format:

$:inkscape --without-gui -f CORE.svg --export-png CORE.png -d 300 -D

$:inkscape --without-gui -f SOFT_CORE.svg --export-png SOFT_CORE.png -d 300 -D

$:inkscape --without-gui -f SINGLETONS.svg --export-png SINGLETONS.png -d 300 -D

**A.2. GenBank formatted published NCBI_PGAP annotation files**

Retrieve genomes of interest and associated metadata from the NCBI:

$:ncbi-genome-download --genera Brachyspira hyodysenteriae bacteria -n > Bhyo-ID.txt

$:ncbi-genome-download --genera Brachyspira hyodysenteriae bacteria --metadata Bhyo-metadata.txt -s genbank

Anvi-script-process-genbank-metadata -m Bhyo-metadata.txt -o Bhyo-NCBI-GENOMES –output-fasta-txt Bhyo-fasta.txt

Snakemake workflow from assembly.fa to contigs.db

Prepare config-contigs.json: add Bhyo-fasta.txt in .json file

$:anvi-run-workflow -w contigs -c config-contigs.json –additional-params –jobs 6 –resources nodes=6

- Prepare an external-genomes.txt (2 columns file: sample name and contigs_db_path). Use metadata information to provide extra information.

$:anvi-gen-genomes-storage -e external-genomes.txt –gene-caller NCBI_PGAP -o GENOMES.db

$:anvi-pan-genome -g GENOMES.db -n Pangenome --mcl-inflation 10 -T7

$:anvi-display-pan -p Pangenome/*PAN.db -g GENOMES.db

$:anvi-split -p Pangenome/*PAN.db -g GENOMES.db -C default -o SPLIT_PANs

$:anvi-display-pan -p SPLIT_PANs/CORE/PAN.db -g GENOMES.db

$:anvi-display-pan -p SPLIT_PANs/SOFT_CORE/PAN.db -g GENOMES.db

$:anvi-display-pan -p SPLIT_PANs/SINGLETONS/PAN.db -g GENOMES.db

**B. Genome similarities**

Computation of average nucleotide identity (ANI analysis):

$:anvi-compute-genome-similarity -e external-genomes.txt -o ANI -p Pangenome/*PAN.db -T7
