# Supplementary figures and images for: Whole-genome analyses reveal a novel prophage and cgSNPs-derived sublineages of Brachyspira hyodysenteriae ST196
Source: BMC Genomics. 2022 Feb 15;23:131. doi: 10.1186/s12864-022-08347-5 (PMC8845278; doi:10.1186/s12864-022-08347-5)

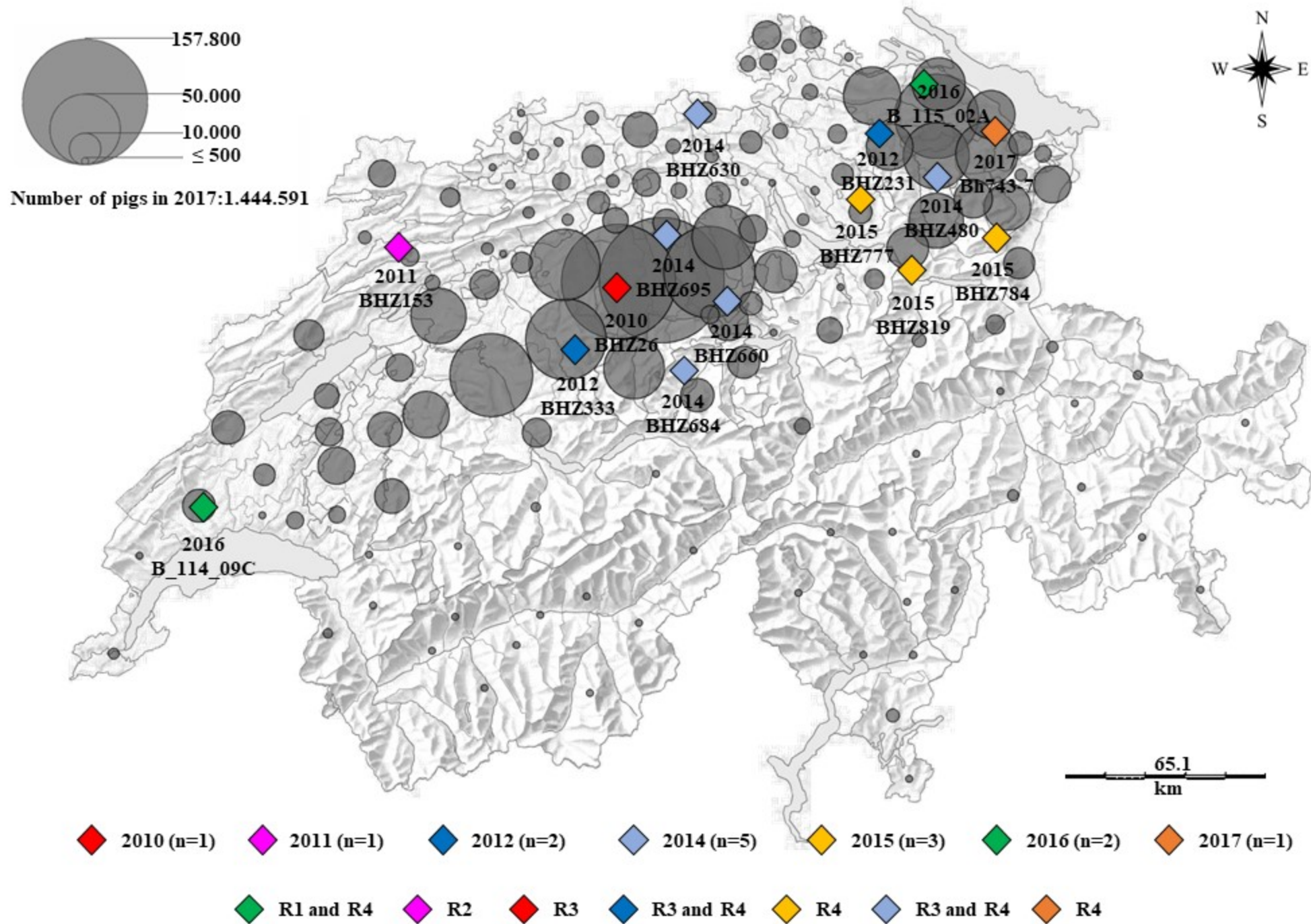

Supplement: Supplementary file 8 — Additional file 8: Figure S1. Geographical distribution of B. hyodysenteriae isolates across Switzerland overtime. Each diamond represents one isolate sampled per herd. Different colors are used to identify samples obtained from geographically distant Swiss pig herds in different years between 2010 and 2017. The basemap indicating density of pig population (grey rings) was obtained from the Federal Statistical Office (http://www.bfs.admin.ch), ThemaKart. [file 12864_2022_8347_MOESM8_ESM.pdf]

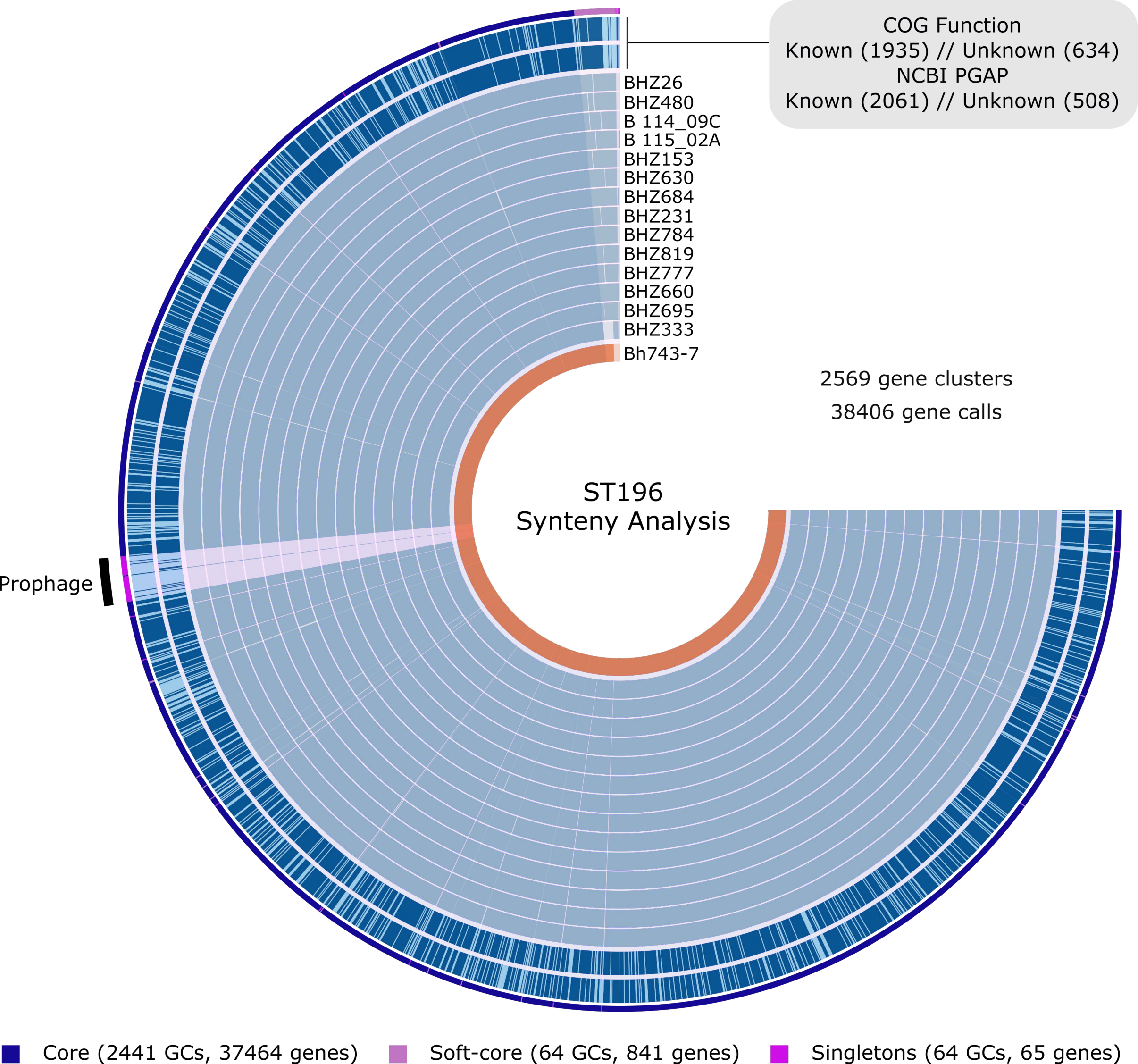

Supplement: Supplementary file 9 — Additional file 9: Figure S2. Synteny analysis. The two most outer layers represent both functional annotations, derived from the COGs database and NCBI-PGAP, and number of genes with either known (dark blue) or unknown (light blue) annotations are also shown. Genomes are organized according to the synteny of the reference genome (in orange). The genome of the novel prophage consisting of phage-like genes organized sequentially and integrated into the chromosome is highlighted in black. Total number of gene clusters (GCs) and genes are indicated for core (dark blue), soft-core (light pink) and singleton (dark pink) bins. [file 12864_2022_8347_MOESM9_ESM.pdf]

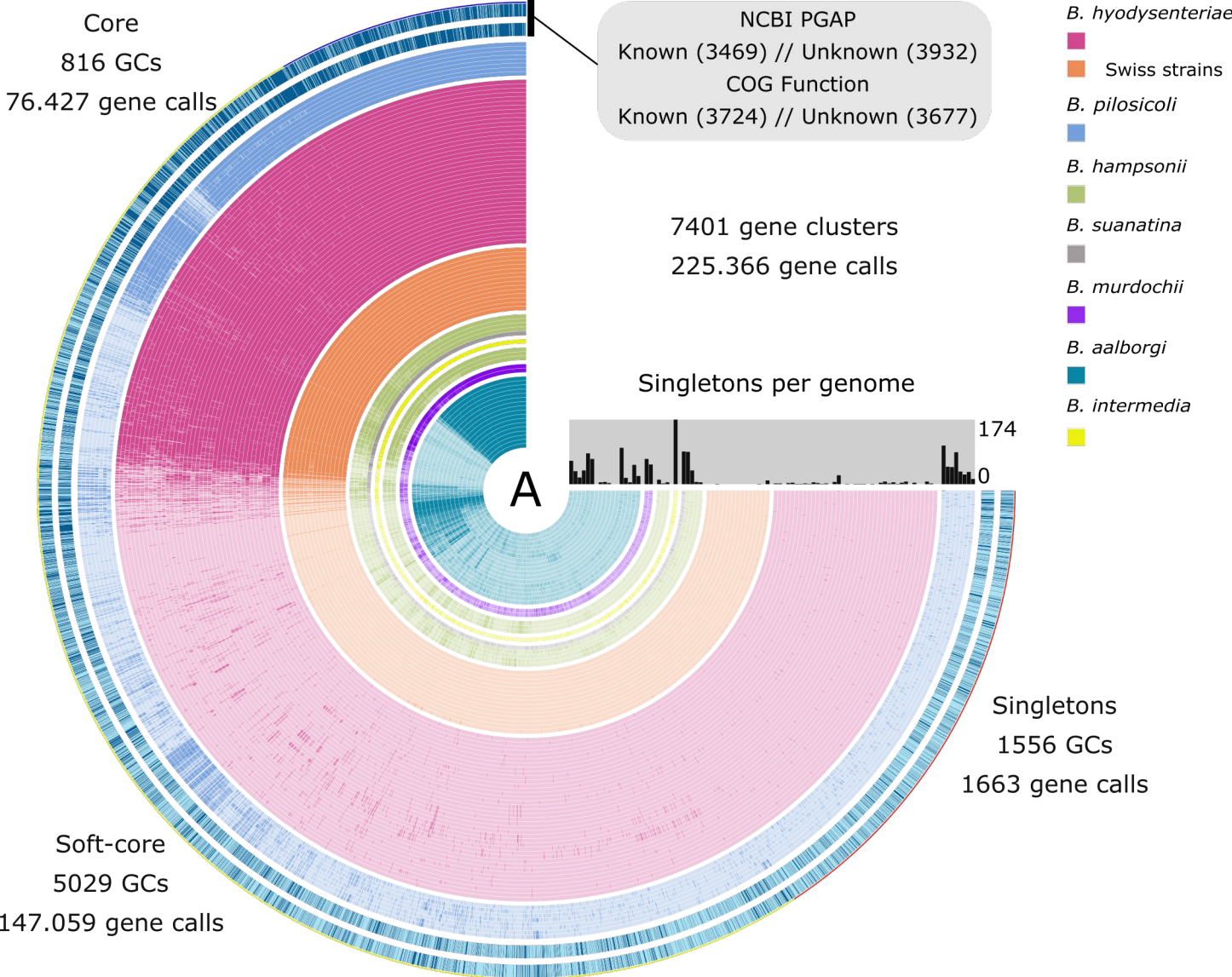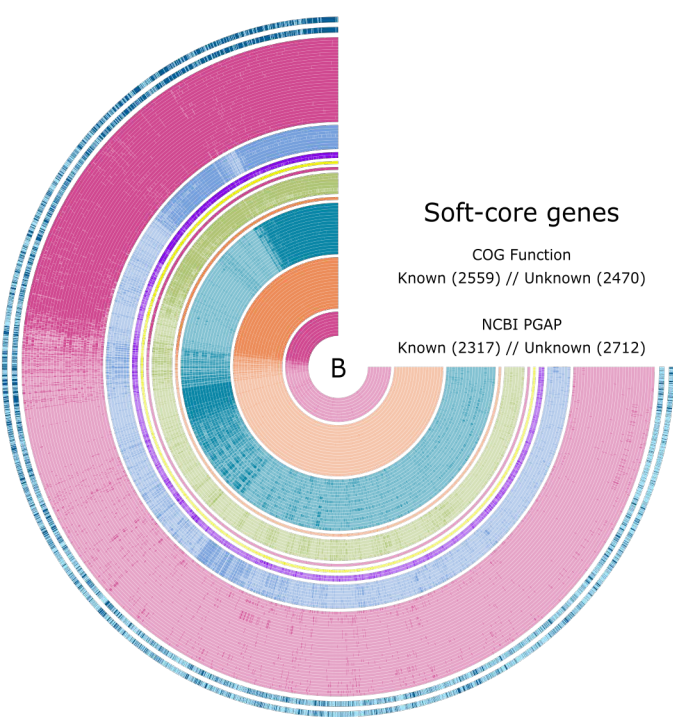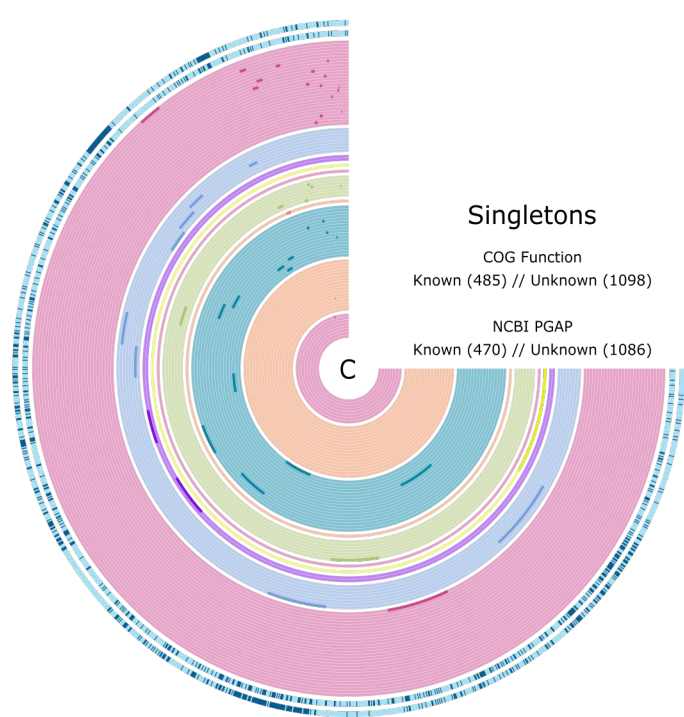

Supplement: Supplementary file 10 — Additional file 10: Figure S3. Pangenome analysis of genomes of different Brachyspira species. (A) General organization and visualization of 90 Brachyspira genomes based on the presence/absence of genes and their contribution to the bins core, soft-core and singleton. Total number of gene clusters (GCs) as well as the number of gene calls falling into each bin are shown in brackets. A graph bar displaying the number of singletons per genome, varying from 0 to 174, is also included.The two outmost layers represent both functional annotations derived from the NCBI-PGAP and COGs database. Known and unknown functions are in dark and light blue, respectively. Total number of both known and unknown functions are indicated in brackets. Genomes are colored-coded according to the seven different species they belong to except Swiss B. hyodysenteriae genomes that are colored in orange to facilitate their visualization. (B) Visualization of the soft-core gene clusters containing a high number of accessory genes. (C) Singletons present in each genome are highlighted and information regarding functional annotation is indicated in brackets. [file 12864_2022_8347_MOESM10_ESM.pdf]
