# Supplementary material for: Whole-genome analyses reveal a novel prophage and cgSNPs-derived sublineages of Brachyspira hyodysenteriae ST196
Source: BMC Genomics. 2022 Feb 15;23:131. doi: 10.1186/s12864-022-08347-5 (PMC8845278; doi:10.1186/s12864-022-08347-5)
Supplement: Supplementary file 8 [file 12864_2022_8347_MOESM12_ESM.docx]

Table 1. Metrics and genetic features of the *de novo* draft assemblies of *B. hyodysenteriae* isolates of ST196

Isolate Name	Metrics[a]											Genetic Features[b]		
	Total contigs	Contigs (>= 500 bp)	Plasmid[c] (bp)	Largest contig (bp)	Total length (bp)	Total length (>= 1000 bp)	N50	N75	L50	L75	G+C (%)	Total CDSs	Coding genes	Total RNAs
B 114_09C	33	32	ND	551051	3034821	3032135	274650	120170	4	7	27.01	2599	2577	40
B 115_02A	28	25	32604	690312	3034997	3032739	411827	120169	3	6	27.01	2598	2575	40
BHZ26	31	31	32555	551026	3039847	3037790	274650	109905	4	8	27.02	2599	2578	40
BHZ153	73	27	32557	550993	3038988	3036730	274650	192471	4	7	27.02	2601	2582	40
BHZ231	58	35	32589	411838	3039186	3036296	265791	100615	5	10	27.02	2604	2583	40
BHZ333	26	26	ND	551023	3002028	2998227	274641	212214	4	7	27.06	2607	2567	40
BHZ480	27	27	31271	658253	3036069	3034114	411835	120189	3	6	27.02	2602	2579	40
BHZ630	33	33	32625	548730	3034298	3032560	274650	102184	4	10	27.02	2601	2582	40
BHZ660	35	34	32600	551004	3035761	3032135	274638	120190	4	8	27.01	2603	2584	40
BHZ684	61	34	32595	551014	3040663	3037144	274631	111522	4	8	27.02	2602	2582	40
BHZ695	33	33	32563	551001	3037330	3034431	274638	107139	4	8	27.02	2601	2581	40
Bh743-7	36	36	32528*	550869	3074392	3069294	273292	100577	4	10	27.02	2664	2639	40
BHZ777	31	31	35553	551017	3036731	3033207	274638	120190	4	7	27.02	2602	2580	40
BHZ784	120	28	32595	551025	3039215	3036957	274650	109908	4	8	27.02	2601	2581	40
BHZ819	31	31	32595	551021	3037581	3034868	274638	120190	4	7	27.02	2601	2580	40

[a]Metrics were obtained from Quast v4.6.0.

[b]Genetic features information taken from the output files of the NCBI-PGAP pipeline annotation.

[c]ND: Plasmids were not obtained from single contigs; *The plasmid pBh743-7 of *Brachyspira hyodysenteriae* isolate Bh743-7 was used as a reference.

Accepted manuscript
